# Supplementary material for: ORN: Inferring patient-specific dysregulation status of pathway modules in cancer with OR-gate Network
Source: PLoS Comput Biol. 2021 Apr 5;17(4):e1008792. doi: 10.1371/journal.pcbi.1008792 (PMC8049496; doi:10.1371/journal.pcbi.1008792)
Supplement: Attachment — Submitted filename: report.pdf [file pcbi.1008792.s044.pdf]

In this work, the authors aim to develop a novel framework to better assess disturbed pathways from patient omics data. Inferring pathway activities from multi-omics profiles is certainly a very relevant goal. However, the reader is hardly convinced by the proposed procedure, mainly due to the lack of clarity concerning the technical procedure.

In particular, the Materials and Methods section would need to be substantially revised. Moreover, it seems to me that the term "pathway" is not appropriate, and that it would be more correct to refer to "sets of molecular components", or rather "upstream modules" as mentioned in the discussion. Indeed, pathways refer to organised networks, with interacting components, and here the authors are able to rather obtain sets of altered components that would explain differential expression.

Major comments

Introduction

- §2: "For example, PARADIGM [4] adapted known pathways as Markov networks and inferred pathway activities from the observed multi-omics profiles with the EM algorithm. " This description needs clarification. I am not sure about the term Markov networks. What is the "EM algorithm"?

  More generally, the introduction lacks a more complete overview of related work and appropriate references.

- §4 (starting "In this work, we present OR-gate Network (ORN)..."): This presentation of the so-called OR-gate networks is too fuzzy. If I understand correctly, an ORN has 3 layers, one including the genes/proteins participating to the signalling pathway, the second being the gate, the third providing the status of the target gene (i.e. deregulated genes).

  "Each layer in ORN is connected by logical OR gates such that the output of a node is true if any input to the gate is true." This is the obvious definition of an OR gate. I do not see why it would handle mutual exclusivity perturbation patterns, as those would rather require a XOR gate.

Material and Methods

- Data preprocessing section: the choice of the range [-2,2] for the CNV booleanisation would deserve a justification. Please also explain the meaning of SGA matrix (fig 2). Similarly, for the parameters used for the booleanisation of the transcriptomic data.

- Leaky OR-gate section: 1st formula is incomprehensible. Notation should be clarified: $X_1 \to y$ means that "X_1 has an effect on y". Later, there is a confusion between Y and y... define δ as $\delta_i = PR(X_i \to y)$.

Furthermore, if I understand well, in Fig 3 the values indicated on the level X correspond to the $Pr(X_i \to y)$, whereas $Pr(X_i = 1)$ will be given by the observation (it is thus not really a probability)...

Anyway, because it seems that this framework is closely relate to the "noisy-OR gates" of ref 19, I would suggest to cite this reference here, and use the description from this reference: (1) each of the causes X_i has a probability $Pr(X_i \to y)$of being sufficient to produce the effect in the absence of all other causes, and (2) the ability of each cause being sufficient is independent of the presence of other causes.

Then, the differences with noisy-OR gates should be better clarified, for which the $Pr(X_i = 1)$ are the numbers labelling the edges in fig 3 B?

Note that the leaky parameter is not a novelty as this was already considered in ref 19.

- OR-gate network section: this section is particularly obscure to me.

  How pathways (and thus their number P) are inferred? The box "ORN inference of fig 2, needs explanations. How matrix U is determined? does it corresponds to the probability Pr(mutation, diff expression)?

  Then, the necessity for optimizing the ORN needs to be explained, I suppose that this is to get the probabilities $Pr(X_i \to y)$, but mostly to get the so-called pathways?

  I was not able to check the remaining of the section as μ and ζ were not defined... Explanations are needed.

- Simulation and evaluation section:

  "Synthetic data was generated by following the noisy OR gate process." I t was said previously that the framework was not based on noisy OR gate... In any case, what is the "noisy OR gate process"?

  "To simulate the mutual exclusivity patterns observed in real data, we performed post pruning. That is, when several mutations belonging to the same pathway took place in the same sample, all but one of them were removed." I have several concerns with this statement. First, it means that mutual exclusivity is supposed to systematically appear in the data, and this is not always the case, as co-occurrences alterations also frequently occur. Furthermore, I wonder how cross-talk are handled with this framework as it can be the case that e.g. for some pathway you would expect exclusivity of alteration for two genes, but for other two other independent pathways this exclusivity is not expected...

- Causal relation extraction section: here again (as indicated before) I wonder how pathways are defined? I suppose that they are derived from the previous procedure, but this is not clear at all. Furthermore, what is the rational underlying the choice of the number of pathways?

Results

- ORN was effective in recovering OR-gate relationships section: what if the number of pathways is larger than 5?
- ORN detected pathways closely related to patient survival section: fo these pathways correspond to documented pathways (in the literature or databases)?

Minor comments

- Generally, the use of the past tense is often awkward (e.g. "In fact, most methods [14–16] modelled mutual exclusivity with statistical tests.", present tense would be more appropriate).
- Introduction §4: correct the sentence "in this novel model with the mechanism from latent pathway? y activities to transcriptomics"
- Data preprocessing section: real-word datasets → real world

  SNV stands for single nucleotide variations... why calling this "non-silent gene-level simple somatic mutations "

  provide the reference for CBioPortal
- Note that (taken from `https://www.cancer.gov/about-nci/organization/ccg/research/structural-genomics/tcga/using-tcga/citing-tcga`) "TCGA requests that authors who use data from TCGA acknowledge the TCGA Research Network in the acknowledgements section of their work. An example of a proper acknowledgement is:

  "The results <published or shown> here are in whole or part based upon data generated by the TCGA Research Network: https://www.cancer.gov/tcga."
