# Supplementary material for: ORN: Inferring patient-specific dysregulation status of pathway modules in cancer with OR-gate Network
Source: PLoS Comput Biol. 2021 Apr 5;17(4):e1008792. doi: 10.1371/journal.pcbi.1008792 (PMC8049496; doi:10.1371/journal.pcbi.1008792)
Supplement: Attachment — Submitted filename: Response to reviewers_final.docx [file pcbi.1008792.s045.docx]

# Response to Reviewer #1:

*As the authors state and show in Fig. 1, the topology of the network is not represented by an ORN, however, this is the basic characteristic of a network. This does not affect the prediction of the nodes involved in the network, only the naming may not be the best*.

Thanks for your comments. We understand that our illustration may cause confusion between a network of signal transduction and a network of OR-gates. We have changed our illustration in the paragraph 2-3 in page 2 and Fig. 1. In addition, “latent pathway” throughout the manuscript were renamed to “pathway module”.

*The method is designed for the mutual exclusivity perturbation pattern and the test data for the first simulation is pre-pro accordingly. Also, it might be a common pattern, what happens if the situation in the patient differ from this pattern?*

Thank you for your insight. Indeed, there are many other mechanisms not captured in our formulation, such as the co-occurrence pattern. And those mechanisms are more difficult to be modeled for various reasons. We have added discussion on this topic in paragraph 3 in Discussion.

In addition, although OR-gate agrees with mutual exclusivity, the most important property of perturbation pattern for single pathways, it is not exclusively designed for this pattern. The fundamental rationale is that mutations affecting the same pathway should have caused similar profiles of differential expression.

*If two different pathways are affected by different mutations but in the same patient, is the method able to assign the DEGs to these pathways or are they merged to a set of affected nodes? In the results different pathways are discussed, in the methods it should be explained a bit more*

Thanks for your insight. Our model usually assigns or partitions DEGs for two pathways according to the information of associations between mutations (or copy number alterations) to perturb a pathway and expression changes of genes regulated by the pathway. Unless these two pathways are co-perturbed in almost all tumors in our data set, our model can distinguish genes regulated by different pathways even they are co-differentially expressed in some tumors. In cases where multiple pathways were dysregulated in the patients, it is likely that estimated pathway modules correspond to a disease mechanism coordinated by multiple pathways. We have added sentences to provide more explanation in the Discussion section( paragraph 4 in page 17).

*It is understandable to use for the neuronal network the same number of layers. However, different methods may need different number of layers for the best results. How do the results from the NN change if more layers are used?*

Thanks for your suggestion. If more layers are added to neural network, it would certainly approximate the OR-gate function or other biological mechanisms in tumors better. However, extracting patterns from a complex neural network is difficult. We have not developed a way to extract latent pathways from a multiple-layer neural network. The main advantage of ORN over traditional neural networks is that it is much easier to catch the mutual exclusivity of pathway perturbation patterns in tumors while integrating the information between pathway perturbation and expression changes of genes.

# Response to Reviewer #2:

*The ORN method of the paper is new; it can be applied to cancer genomic and transcriptomic data, to infer latent pathway activities. The approach can potentially contribute a lot to the field of cancer pathway studies. It will be more convincing to have additional testing datasets of other cancers.*

Thanks for your comment, we have added another experiment on liver cancer in the result section (paragraph 4 in page 9). Results showed that two pathways were related to patient survival (paragraph 1-2 in page 13, and Fig. 8)

*Page 6, the sentence seems incomplete: "Furthermore, in this novel model with the mechanism from latent pathway y activities to transcriptomics, a pathway perturbed in a subset of patients cause differential expression in a subset of genes."*

Thanks for your feedback. The paragraph with this sentence has been rewritten (paragraph 4 in page 2).

# Response to Reviewer #3:

*It seems to me that the term ”pathway” is not appropriate, and that it would be more correct to refer to ”sets of molecular components”, or rather ”upstream modules” as mentioned in the discussion.*

Thanks for your suggestion. We have changed the “pathways” to “pathway modules”.

*”For example, PARADIGM [4] adapted known pathways as Markov networks and inferred pathway activities from the observed multi-omics profiles with the EM algorithm. ” This description needs clarification. I am not sure about the term Markov networks. What is the ”EM algorithm”?*

*More generally, the introduction lacks a more complete overview of related work and appropriate references.*

Thanks for your comment. We have removed the technical details in the description of PARADIGM. As for related works, a few studies were added. Still, we are not able to find many studies aiming at inferring patient-specific pathways with multiple omics data. Thus, we added a paragraph about studies investigating pathways from only transcriptomics, so as to provide a more complete background. (Paragraph 2 in page 1)

*(starting ”In this work, we present OR-gate Network (ORN)...”): This presentation of the so-called OR-gate networks is too fuzzy. If I understand correctly, an ORN has 3 layers, one including the genes/proteins participating to the signalling pathway, the second being the gate, the third providing the status of the target gene (i.e. deregulated genes)*

Thanks for your feedback. Yes, our ORN has 3 layers. The input layer is for somatic genomic alterations (mutations and copy number alterations) of genes for tumors. The hidden layer is for pathway or upstream modules. The output layer is for expression status of genes for tumors. We have modified the description in paragraph 5 and 6. We also added description at the beginning of Overview in Methods.

*Data preprocessing section: the choice of the range [-2,2] for the CNV booleanisation would deserve a justification. Please also explain the meaning of SGA matrix (fig 2). Similarly, for the parameters used for the booleanisation of the transcriptomic data*

Thanks for your feedback. The SGA matrix represents whether SNV or CNV took place in a gene in a certain sample. The choice of range for CNV was decided because values of 1/-1 was less correlated with differential expression. The parameters in transcriptomic data processing is to identify individual DEGs with the criteria of P<0.05. We have added brief notes in paragraph 2 and 3 in the Data preprocessing section.

*”Each layer in ORN is connected by logical OR gates such that the output of a node is true if any input to the gate is true.” This is the obvious definition of an OR gate. I do not see why it would handle mutual exclusivity perturbation patterns, as those would rather require a XOR gate.*

Thank you for your comment. Mutual exclusivity is more of a statistical phenomenon than a strict logical rule [1-3]. It is biologically possible that a few tumors may have more than one SGAs to perturb a pathway. XOR gates would be too stringent for our applications.

The statistical rational underlying mutual exclusivity is the “collider” shapes in the Bayes network formulation. That is, multiple independent causes become negatively correlated given that their common effect occurs. By connecting SGAs to pathway status with OR-gates, SGAs become conditionally dependent given the estimated pathway status. This induced the mutual exclusivity pattern to emerge in the OR-gate network.

*Furthermore, if I understand well, in Fig 3 the values indicated on the level X correspond to the P r(Xi → y), whereas P r(Xi = 1) will be given by the observation (it is thus not really a probability)... Anyway, because it seems that this framework is closely relate to the ”noisy-OR gates” of ref 19, I would suggest to cite this reference here, and use the description from this reference**: (1) each of the causes Xi has a probability P r(Xi → y)of being sufficient to produce the effect in the absence of all other causes, and (2) the ability of each cause being sufficient is independent of the presence of other causes. Then, the differences with noisy-OR gates should be better clarified, for which the P r(Xi = 1) are the numbers labelling the edges in fig 3 B? Note that the leaky parameter is not a novelty as this was already considered in ref 19.*

Thank you for your comment. When OR-gate is used between latent pathway and DEGs, X is not fixed because the status of latent pathways is uncertain. That is the major difference between our model and the original version. We have added the description from the original reference (Paragraph 3 in page 5). Fig. 3 has been removed because we misunderstood the original paper. We also added that “we followed the original work to introduce the leaky parameter” (paragraph 6 in page 5).

*OR-gate network section: this section is particularly obscure to me. How pathways (and thus their number P) are inferred? The box ”ORN inference of fig 2, needs explanations. How matrix U is determined? does it corresponds to the probability P r(mutation, diff expression)? Then, the necessity for optimizing the ORN needs to be explained, I suppose that this is to get the probabilities Pr(Xi → y), but mostly to get the so-called pathways? I was not able to check the remaining of the section as µ and ζ were not defined... Explanations are needed.*

Sorry for the confusion. Essentially U and Z were determined by identifying the maximum likelihood of the ORN regarding $\mu$ and $\zeta$. Then U and Z can be recovered with the sigmoid transformation of $\mu$ and $\zeta$. We have restructured the “OR-gate network” section and added a section “Gradient-based model estimation” focusing on model estimation with the illustration of pseudo code.

µ and ζ were introduced so we can estimate U and Z by unconstrained gradient descent (the last paragraph in page 6). Explanation regarding µ and ζ were added. The section “causal relation extraction” was placed right after model estimation to explain how to extract U, Z, and pathway activities in more detail.

*Simulation and evaluation section: ”Synthetic data was generated by following the noisy OR gate process.” It was said previously that the framework was not based on noisy OR gate... In any case, what is the ”noisy OR gate process”?*

Thank you for your comment, we changed the term “process” to “mechanism”. It simply refers to putting synthetic mutation data through the probabilistic OR-gate layers described in the Method section.

*”To simulate the mutual exclusivity patterns observed in real data, we performed post pruning. That is, when several mutations belonging to the same pathway took place in the same sample, all but one of them were removed.” I have several concerns with this statement. First, it means that mutual exclusivity is supposed to systematically appear in the data, and this is not always the case, as co-occurrences alterations also frequently occur. Furthermore, I wonder how cross-talk are handled with this framework as it can be the case that e.g. for some pathway you would expect exclusivity of alteration for two genes, but for other two independent pathways this exclusivity is not expected...*

Simulation experiments were merely designed to evaluate whether the ORN model can infer ground truth when the data is generated through the process assumed by the model. As you pointed out, mutual exclusivity is not guaranteed to emerge from the OR-gate mechanism. So we took one step further to evaluate whether ORN can handle mutual exclusivity as we expected.

Our model not only takes care of the mutual exclusivity of pathway perturbations in tumors, but also captures the causal relation between pathway perturbations (by gene mutations) and expression changes of genes regulated by the perturbed pathway. In some tumors, two or more genes on the same pathway may have mutated together. These mutations can also activate an OR gate that represents the pathway. Suppose mutations of two different genes are mutually exclusive, if their mutations are associated with the expression changes of the same set of genes, our model considers these two genes to be on the same pathways; else we consider that the mutations of these two genes perturb two independent pathways. Some other models [1-3] that only consider the mutual exclusivity of somatic genomic alterations (SGAs) may have the problem you mentioned: if two genes with mutually exclusive SGAs are on the same pathway or two independent pathways. This has been discussed in paragraph 2 in Discussion.

*Causal relation extraction section: here again (as indicated before) I wonder how pathways are defined? I suppose that they are derived from the previous procedure, but this is not clear at all.*

Latent pathways (renamed as pathway modules) and their patient-specific status are hidden variables in ORN to be inferred. Although we hope it corresponds to real biological pathways, it may capture something else. We have further clarified this topic in paragraph 2 in page 17.

Since we did not use domain knowledge, the identity of latent pathways can only be analyzed after parameter estimation. After the ORN is trained, after removing edges with weights smaller than the threshold, nodes/genes (in the input layer) that are connected to a node in the hidden layer are defined as a pathway module. We have restructured this Section to clarify the procedure.

*Furthermore, what is the rational underlying the choice of the number of pathways?*

We have tried AIC and BIC in simulation experiments. They underestimated the true number of pathways needed. So the choice of the number of pathways is arbitrary. Still, when real datasets were analyzed with a different number of pathways, the result seems similar. We have added this limitation in our Discussion section. (the end of paragraph 3 in page 18)

*ORN was effective in recovering OR-gate relationships section: what if the number of pathways is larger than 5?*

Thanks for your comment. We have added simulation settings where the number of latent pathways grow larger. It results in ORN becoming more unstable. Fig. 4 was modified to include the evaluation results. During real data analysis, it seems the appropriate number of pathways does not exceed 15. Hence the performance of ORN in real data experiment should be sufficient.

*ORN detected* *pathways closely related to patient survival section: do these pathways correspond to documented pathways (in the literature or databases)?*

Thanks for your question. We used Gene Ontology (GO) to find which biological process is closely related to genes in each pathway module (enrichment analysis). According to GO enrichment analysis, pathway 6 is mostly related to DNA processing activities such as DNA replication. Pathway 7 is more about immune response and virus infection. (paragraph 1-2 in page 12)

*Generally, the use of the past tense is often awkward (e.g. ”In fact, most methods [14–16] modelled mutual exclusivity with statistical tests.”, present tense would be more appropriate).*

Thanks for your comment, we have changed “modelled” and other past tense in Introduction and Method.

*Introduction §4: correct the sentence ”in this novel model with the mechanism from latent pathway? y activities to transcriptomics”*

Thanks for pointing it out. The sentence has been removed

*Data preprocessing section: real-word datasets → real world SNV stands for single nucleotide variations... why calling this ”non-silent gene-level simple somatic mutations ” provide the reference for CBioPortal*

Typos corrected. “simple somatic mutations” was changed to “single nucleotide variations”. Reference for CBioPortal was added.

*Note that (taken from https://www.cancer.gov/about-nci/organization/ ccg/research/structural-genomics/tcga/using-tcga/citing-tcga) ”TCGA requests that authors who use data from TCGA acknowledge the TCGA Research Network in the acknowledgements section of their work. An example of a proper acknowledgement is: ”**The results here are in whole or part based upon data generated by the TCGA Research Network: https://www.cancer.gov/tcga.”*

Acknowledgement to TCGA has been added.

# Reference

1.	Vandin, F., E. Upfal, and B.J. Raphael, *De novo discovery of mutated driver pathways in cancer.* Genome Res, 2012. **22**(2): p. 375-85.

2.	Kim, Y.A., S. Madan, and T.M. Przytycka, *WeSME: uncovering mutual exclusivity of cancer drivers and beyond.* Bioinformatics, 2017. **33**(6): p. 814-821.

3.	Leiserson, M.D., et al., *Erratum to: CoMEt: a statistical approach to identify combinations of mutually exclusive alterations in cancer.* Genome Biol, 2016. **17**(1): p. 168.
