# Supplementary material for: ORN: Inferring patient-specific dysregulation status of pathway modules in cancer with OR-gate Network
Source: PLoS Comput Biol. 2021 Apr 5;17(4):e1008792. doi: 10.1371/journal.pcbi.1008792 (PMC8049496; doi:10.1371/journal.pcbi.1008792)
Supplement: Attachment — Submitted filename: Response to Reviewers 01282021.docx [file pcbi.1008792.s046.docx]

# Response to Reviewer #1 and #2:

Thank you for your approval. In this revision, we have tried to improve the writing of the manuscript. Apart from reviewer #3’s suggestions, we added a paragraph in Introduction to emphasize the computational difficulty to estimate the biologically plausible signalling network. The rest of the changes were related to spelling, tenses, and grammars. The content of methods and experimental results remain the same since the last revision.

# Response to Reviewer #3:

*Unfortunately, this revised manuscript contains too many typos, sentences that are poorly worded and problematic use of the past tense.*

We apologize for your inconvenience caused by spelling and grammatical errors. In this revision, we have checked and corrected numerous errors of spelling, grammar, and tenses throughout the manuscript. We hope you will find the manuscript improved in writing quality.

*1) The definition of a “pathway module” is still lacking and, in my opinion, the concept is still unclear; does it corresponds to the entries of a gate in the ORN (as suggested in Fig2)? It is however briefly discussed in the discussion, which in my opinion is not the right place to explain it.*

Thanks for your question. A pathway module is a group of genes whose somatic alteration dysregulate the same pathway and drive similar phenotypes. We have restructured part of Introduction to add this definition in the 5^th^ paragraph in this section. In the 2^nd^ paragraph in Causal relation extraction, we also explained “pathway module” in light of how we extracted them from ORN.

*2) Figure 1 and related text (paragraphs “In this work, we present OR-gate Network...”, and “Similarly, we use OR-gate to model how pathway...” page 2 including the figure caption) should be seriously revised.*

Thanks for pointing out that the last several paragraphs in Introduction are quite confusing and difficult to read. We have rewritten most of these three paragraphs to better convey how ORN is aligned with the well-known statistical patterns in somatic alteration profiles and gene expression profiles.

*Fig 1A is supposed to represent an example of a signalling cascade (where molecular components are not genes but their products, please correct this in the description), leading to the activation of transcription factors, which in turn controls the transcription of target genes.
Fig1B is misleading as it is. I my opinion, it does not provide support for understanding the ORN model.
a)The inputs of the gate should be drawn differently than the molecular components of the singalling cascades in Fig 1A as they correspond to the genes (whereas in Fig1A, we had their products).
b)I don’t see why the TF would be the gate, rather it should also be an input of the gate (i.e. if CCND1 is mutated, it will affect the expression of its targets...). There should be an OR gate (not corresponding to a molecular component). I suppose that this is the “additional variable indicating the pathway status”?
c)The levels should be named appropriately. What is the genomic level? What is the pathway status (is it given by the output of the gate?)*

Thanks for your comment. We have added SGA events in Fig1A to emphasize the role of SGAs in pathway dysregulation. In Fig1B, the molecular components were replaced by SGA. The TF has been added to the input. And the OR gate output of SGA became the pathway abnormality status (PAS). Naming of the levels were changed to SGA, PAS, and DEG.

*3) Section Probabilistic OR-gate, page 4
All this section requires a complete and carefully revision. In particular, the notation is not coherent, and there are too many typos: Xi (i should be a subscript), capital vs small letter (e.g. for Y or y), bold letter for vector X (in OR(X,delta) but not in the text), first index of X, 0 or 1 (in “and delta represents the vector [Pr(X0->Y...”)... And in the last formula what X_other represents?
Please check lines 9 in Fig 3 (what are the dimensions L and N?).*

Thanks for pointing out the inconsistency among Mathematical notions. We have corrected these notions. In the revised manuscript, $X_{0}$ corresponds to unmodeled cause. X_other was removed. In Fig 3, L and N were changed to P and G.

*4) the equation defining the error needs some explanation. I don’t understand the subscripts sg.*

Thanks for your comment. The first equation in Simulation and evaluation has been corrected.
